# Safety and Efficacy of s-MOX Regimen in Patients with Colorectal Cancer Who Developed Cardiotoxicity Following Fluoropyrimidine Administration: A Case Series

**Published:** 2020

**Authors:** Matthew I. Ehrlich, Kristin Kaley, Melissa Smith, M. Wasif Saif

**Affiliations:** 1 Northwell Health Cancer Institute, Feinstein Institutes of Research and Zucker School of Medicine, Lake Success, New York, USA; 2 Yale Cancer Center, New Haven, CT, USA; 3 Tufts Cancer Center, Boston, MA, USA

**Keywords:** MOX Regimen, Fluoropyrimidines, Cardiotoxicity, Dihydropyridine Dehydrogenase

## Abstract

**Background:**

Fluoropyrimidines compose the backbone of regimens to treat many common solid tumors, including gastrointestinal (GI), breast and head/neck. As we continue to use these agents routinely, recognition of rare but real toxicities, such as cardiotoxicity, has also improved. The treatment options for patients who have encountered fluoropyrimidine-induced cardiotoxicity are limited as many anti-angiogenic drugs also pose a cardiac risk.

**Patients and Methods:**

We present a case series of three patients who developed cardiotoxicity in the form of anginal-like symptoms, EKG changes and elevated cardiac enzymes on infusional 5-FU or capecitabine and were subsequently treated with the s-MOX (simplified-mitomycin-oxaliplatin) regimen for their metastatic colorectal cancer (mCRC). All three patients were tested for polymorphic abnormality of *DYPD* and *TYMS*.

**Results:**

All three patients were treated with s-MOX consisting of mitomycin-C 7 mg/m^2^ on day 1 and oxaliplatin 85 mg/m^2^ on days 1 and 15 (1 cycle = 28 days) after they encountered cardiotoxicity to 5-FU and/or capecitabine. None of these patients developed any cardiotoxicity on s-MOX. Overall, the MOX regimen was well tolerated. The most common toxicities included ≤ 2 grade peripheral neuropathy, nausea, vomiting, thrombocytopenia, and anemia. Grade ≥ 3 toxicities included neutropenia (10%), thrombocytopenia (33%), vomiting (8%), and peripheral neuropathy (30%). *DYPD* gene was normal in all patients and *TYMS* was abnormal (2R/2R) in one patient.

**Conclusion:**

This is the first case series that reports the safety and feasibility of s-MOX in patients with mCRC who developed cardiac toxicity to 5-FU or capecitabine. The s-MOX regimen may provide an alternative treatment option for patients who either develop fluoropyrimidine-related cardiotoxicity or who have abnormalities in the *DYPD* gene.

## Introduction

5-fluorouracil (5-FU), an antimetabolite in the fluoropyrimidine class, is the third most commonly used chemotherapeutic agent worldwide for the treatment of solid malignancies [1]. Despite advances in novel cancer therapies, commonly used in combination with fluoropyrimidines, 5-FU remains one of the most effective and safe chemotherapy agents to manage colorectal cancer (CRC) [2]. The most common toxicities of 5-FU include mucositis, diarrhea, nausea, vomiting, and neutropenia [3]. The syndrome of cardiotoxicity has also been recognized in association with fluoropyrimidines, which are now reported as the second most common chemotherapeutic drug associated with cardiotoxicity after anthracyclines, with an incidence ranging from 1.5% to 18% [4]. Clinical manifestations of cardiotoxicity due to fluoropyrimidines may include angina, acute coronary syndrome including myocardial infarction, arrhythmias, heart failure and even sudden death [4]. Fluoropyrimidine-induced cardiotoxicity is known to vary with the method and schedule of administration, and dose of drug. While the mechanism by which these drugs provoke cardiac toxicity is largely unknown, several theories have been postulated; these include coronary vasospasm, increased endothelial thrombogenicity, direct toxic effects on endothelial cells, autoimmune reactions and variations in drug metabolism [5–7]. Abnormalities in the enzyme dihydropyridine dehydrogenase (*DYPD*) have also been associated with severe cardiotoxicity in patients taking fluoropyrimidines [5,6]. Anecdotal reports have also found this toxicity in patients with thymidylate synthase (TYMS) genetic polymorphisms [8].

The treatment options for patients who have encountered fluoropyrimidine-induced cardiotoxicity may include switching to a different fluoropyrimidine, switching to a different schedule of intravenous 5-FU or switching to a non-fluoropyrimidine-containing chemotherapy regimen if one exists [1,9]. The former two options still carry an unacceptably high rate of cardiotoxicity. Non-fluoropyrimidine options consist of irinotecan alone or in combination with cetuximab, cetuximab alone, panitumumab alone, regorafenib or immunotherapy for patients with MSI-high colorectal cancer [10–15]. Unfortunately, these alternatives are not without their own toxicities. Similar to the fluoropyrimidines, drugs such as anti-VEGF inhibitors (i.e., bevacizumab, aflibercept, regorafenib) have also been reported to cause cardiotoxicity in patients being treated for colorectal cancer [16]. The one exception in the literature appears to be raltitrexed, a non-fluoropyrimidine thymidylate synthase inhibitor, which was found to be safe in patients who had previously experienced cardiotoxicity on fluoropyrimdines [17–18].

Mitomycin-C represents another potential option for patients with mCRC who are intolerant of fluoropyrimidines. Mitomycin-C is a natural antibiotic isolated from *Streptomyces caespitosus* or *Streptomyces lavendulae* that displays antitumor activity [19]. Although it is not commonly used for CRC, mitomycin-C does offer modest activity as shown in previous studies [20–23]. Similarly, oxaliplatin has been shown to have activity against mCRC refractory to 5-FU, both as a single-agent and in combination with fluoropyrimidines [24].

In order to treat patients who either developed cardiac toxicity to fluoropyrimidines, were found to have *DYPD* deficiency, or were intolerant to fluoropyrimidines, we developed a simplified regimen combining mitomycin-C with non-fluoropyrimidine agents, such as oxaliplatin, and named it s-MOX. In this paper, we describe a case series of three patients who were treated successfully and safely with s-MOX consisting of mitomycin-C 7 mg/m^2^ on day 1 and oxaliplatin 85 mg/m^2^ on days 1 + 15 (1 cycle = 28 days) after they encountered cardiotoxicity to 5-FU and/or capecitabine.

## Patients and Methods

Pharmacy records were used to indicate patients who were treated with the s-MOX regimen for mCRC from July 2012 - March 2019. Review of electronic charts was performed. When a cardiac event was identified in a patient on a fluoropyrimidine, the paper chart was searched for details as outlined below. Every effort was made to review laboratory tests, including EKG, MUGA scan and reports of stress test if performed. Two patients were identified from our clinic, while one patient included in this study was referred to us from China after relocating to Boston for a second opinion. Cardiotoxicity was defined as angina-like symptoms including chest pain, shortness of breath, palpitations, abnormal cardiac enzyme results, ischemic changes or arrhythmia on EKG, abnormal stress test or cardiac catheterization.

The data including age, sex, diagnoses, prior treatments, dose of fluoropyrimidine, predisposing risk factors, presenting symptoms, laboratory results including cardiac enzymes, EKGs, echocardiograms, stress tests and cardiac catheterizations were collected. Risk factors for ischemic heart disease were smoking, diabetes, hypertension, hypercholesterolemia, and family history of ischemic heart disease. Information on medical therapy for cardiac symptoms was also obtained. For patients rechallenged with fluoropyrimidines, presenting symptoms after this treatment were recorded as well.

Two patients were tested for polymorphic abnormality of *DYPD* with TheraGuide 5-FU™ (Myriad Genetic Laboratories, Inc., Salt Lake City, UT, USA) pharmacogenetic test and the third patient was tested at Dr. Diasio’s laboratory with RT-PCR as previously published [25]. In addition, we also collected specimens to analyze polymorphisms associated with *TYMS* as described previously [8].

## Results

A total of 13 patients received the s-MOX regimen in the palliative setting for mCRC due to failure and/or intolerance of previous regimens. We identified that three of these 13 patients had encountered fluoropyrimidine-associated cardiotoxicity. The demographic characteristics of these patients, as well as the treatment regimens and cardiac-related symptoms, are presented in Table 1.

All three patients had received some form of chemotherapy prior to the presenting study. First-line treatment in these patients were: patient 1 had received IFL regimen containing 5-FU/leucovorin and irinotecan, patient 2 received FLOX regimen containing 5-FU/leucovorin and oxaliplatin, and patient 3 received S-1 regimen containing ftorafur, gimeracil and oteracil (patient 3 was referred to us from China). Second-line treatment in these patients upon progression included: patient 1 was treated with FOLFOX-6 containing 5-FU 2400 mg/m^2^/46h along with oxaliplatin 85 mg/m^2^ and leucovorin 400 mg/m^2^, patient 2 was treated with FOLFIRI containing 5-FU 2400 mg/m^2^/46h along with irinotecan 150mg /m^2^ and leucovorin 400 mg/m^2^, while patient 3 received capecitabine 850 mg/m^2^ with xaliplatin 100 mg/m^2^ on day 1 every 3 weeks.

Symptoms in all three patients included angina-like chest pain as well as some combination of dyspnea, palpitations, nausea and diaphoresis. EKG revealed supraventricular tachycardia in patient 1, sinus tachycardia with hyperacute T waves in the anterolateral leads in patient 2 and normal sinus rhythm with T wave inversion in leads III and VI in patient 3. All patients had troponin elevations (range 0.09 ng/ml - 0.25 ng/ml). All three patients underwent additional diagnostic tests. Patient 1 had an adenosine stress test showing LVEF of 68% and normal wall motion and perfusion. Patient 2 had a MIBI/sestamibi scan using technetium (^*99m*^*Tc*) *which showed LVEF of 68% and normal perfusion. Patient 3 underwent cardiac catheterization revealing moderate coronary artery disease with 40–50% disease of the left coronary, and an echocardiogram showing LVEF of 55% and left ventricular hypertrophy*. Patients 1 and 3 were placed on medical therapy for their cardiac symptoms, while patient 2 was not. Patient 1 received metoprolol 12.5 mg bid, oral aspirin 81 mg once daily and sublingual nitroglycerin as needed for chest pain. Patient 3 was started on Toprol XL 25 mg once daily, atorvastatin 10 mg once daily and oral aspirin 81 mg once daily. *DYPD* gene testing was normal in all three patients. *TYMS* was abnormal (2R/2R) in patient 2. Of the three patients, only patients 1 and 2 were rechallenged with capecitabine, resulting in recurrent substernal chest pain.

Three patients treated with the s-MOX regimen were found to have mutations in KRAS, making them ineligible for cetuximab or panitumumab. TAS-102 was not approved during the treatment time of these patients [26,27].

Overall, the s-MOX regimen was well tolerated. Twelve percent of patients required dose reductions and eight percent experienced treatment delays. The most common toxicities included ≤ grade 2 peripheral neuropathy, nausea, vomiting, thrombocytopenia, and anemia. Grade ≥ 3 toxicities included neutropenia (10%), thrombocytopenia (33%), vomiting (8%), and peripheral neuropathy (30%).

The best overall response was stable disease in these patients, with a median duration of response of 4 months (range: 3–6.5). Median time to treatment failure (TTF) was 5.6 months (range: 4–7).

## Discussion

This is the first case series that reports the safety and feasibility of s-MOX in patients with mCRC who developed cardiac toxicity to 5-FU or capecitabine. Our data shows that mitomycin-C combined with oxaliplatin is feasible and offers modest therapeutic index in patients with fluoropyrimidine/leucovorin-pretreated mCRC with stable disease, with a median duration of response of 4 months and median TTF of 5.6 months. We searched the literature and found only one published study detailing the use of the s-MOX regimen in same patient population [21]. In this study, 64 patients with mCRC who had progressed on a fluoropyrimidine-based regimen were randomized to receive either MOX or MIRI (mitomycin-C plus irinotecan). Both regimens provided an acceptable therapeutic index. The major side effects of the MOX regimen included neutropenia (68%), thrombocytopenia (81%), emesis (52%) and peripheral neuropathy (48%). Our study showed reduced toxicities, probably due to a reduced dose of mitomycin and upfront use of antiemetic per NCCN guidelines.

Mitomycin-C is often considered an antiquated drug, not commonly used due to its associated delayed bone marrow toxicity and potential to cause hemolytic uremic syndrome (HUS), lung fibrosis and renal damage [28,29]. Nonetheless, various studies have found success using this drug in the treatment of tumors of the gastrointestinal tract (anal, upper gastrointestinal), breast, lung and bladder [20]. In the setting of salvage treatment for mCRC, data does not support the use of single agent mitomycin; however, combinations such as MIXE (mitomycin with capecitabine), MIRI and MOX have shown modest activity [21–23]. While cardiotoxicity is not very commonly associated with mitomycin-C, several studies have reported a relationship. One prospective study of 44 patients treated with mitomycin-C reported that one patient developed cardiac failure as measured by a drop-in ejection fraction after 30 mg/m^2^ of the drug and only 150 mg/m^2^ of doxorubicin. Based on the literature and this study, these authors posited that cardiotoxicity is dose-dependent, occurring at doses of 30 mg/m^2^ or more, mainly in patients receiving either past or concurrent anthracyclines [30]. Another study of 91 patients with advanced breast cancer reported that 15.3% of patients receiving mitomycin-C developed symptoms of congestive heart failure, compared to only 3.4% of patients from a similar cohort who did not receive the drug [31]. However, cardiotoxicity has not been reported in patients receiving lower doses than reported by these two studies. We selected a 7 mg/m^2^ dose for mitomycin due to our previous experience with the MIXE regimen [20].

Interestingly, all three patients had been treated with fluoropyrimidine-containing regimens prior to developing cardiotoxicity during the present study. While two patients had received only bolus 5-FU previously, one had received S-1. S-1 contains tegafur (FF) and two types of enzyme inhibitors, gimeracil/5-chloro-2,4-dihydroxypyridine (CDHP), a potent inhibitor of DPD, and potassium oxonate (Oxo), which inhibits phosphorylation of intestinal 5-FU in a molar ratio of 1:0.4:1 [32]. S-1 is approved in Japan, China, Taiwan, Korea, Singapore, and several European countries but is not available in the USA. In the published phase II or III studies of S-1, no grade III or IV cardiovascular events were reported [5]. We believe that this patient did not develop cardiac toxicity on S-1 due to the fact that gimeracil is a highly active reversible *DPD* inhibitor; *DPD* inhibition by gimeracil results in significantly reduced levels of cardiotoxic catabolites of 5-FU, hence resulting in less cardiotoxicity. This is further supported by clinical experience with the use of S-1 in CRC patients with previous 5-FU- or capecitabine-induced cardiotoxicity [33,34].

Nevertheless, even alternatives are limited by potential toxicities, including irinotecan, regorafenib and pembrolizumab [12–18]. Previous studies have shown that rechallenging a patient with previous 5-FU cardiotoxicity with either a lower dose or a different mode of administration could result in repeat cardiac complication or even death [35]. Recurrence rates may be as high as 90%, and one systematic review of the published literature reported a 13% fatality rate [5,36^].^ Prophylactic measures such as administering nitrates or calcium channel blockers have not been found to be uniformly effective [5]. Thus, rather than rechallenge such patients, using a non-fluoropyrimidine-containing regimen may be an alternative option.

All three of our patients tested negative for abnormalities in the *DYPD* gene. However, we have previously published cases of patients with DPD deficiencies who developed severe toxicities after receiving fluoropyrimidine-based therapies [5,37,38]. As a response to this issue, the European Society for Medical Oncology changed their guidelines earlier this year to reflect the potential for severe toxicities from fluoropyrimidines in such patients. The society now recommends testing all patients who will be receiving 5-FU, and the related medications capecitabine and tegafur, for *DPD* deficiency prior to starting treatment [39]. As such, it is now increasingly important to find alternatives to fluoropyrimidine-containing regimens for patients who will be unable to tolerate these drugs. Hence, this study offers another option for these patients.

This case series is limited by the small sample size of patients. As cardiotoxicity is a relatively uncommon side effect of fluoropyrimidines, few patients at our clinic met criteria for this retrospective analysis.

*In summary*, while fluoropyrimidines have served as the backbone of treatment for mCRC, it has become evident that alternative options are needed. Cardiotoxicity and related side effects can cause life-threatening issues in select patients. We have shown that the s-MOX regimen offers a favorable toxicity profile with an acceptable therapeutic index in patients with mCRC who develop fluoropyrimidine-related cardiotoxicity or who are found to have DPD deficiencies.

## Figures and Tables

**Table 1:** Description of three patients who developed cardiotoxicity associated with 5-FU and later treated with s-MOX.

Patients #	Age/Sex/Race	Diagnosis	Dose of 5-FU/Capecitabine	Previous Therapy	Risk Factors	Symptoms	ECG	Cardiac Markers	Diagnostic Tests	Therapy	Rechal-lenge
1	55/F/C	mCRC	5-FU 2400 mg/m2 over 46 hr. (F0LF0X-6)	IFL	HTN	Substernal chest pain + dyspnea + Palpitations	Supraven-tricular tachycardia	CK 210 MB 3.4, Troponin 0.i9ng/ml	*Adenosine stress test*: LVEF 60% with normal wall motion and normal perfusion	Metoprolol 12.5 mg twice daily ASA 8img once daily SLNTGprn	No
2	39/M/C	mCRC	5-FU 2400 mg/m2 over 46 hr. (FOL-FIRI)	FLOX	Remote Smoking	Substernal chest pain + Nausea + Fatigue	Sinus tachycardia, hyper acute T waves in anterolateral leads	CK56, MB 0.9, Troponin 0.09ng/ml	*MIBI* scan/*sestamibi* scan is using *Technetium (*^*99m*^*Tc)* showed normal perfusion and LVEF 68%	None	Yes, with recurrent substernal chest pain on capecitabine
3	64/M/A	mCRC	Capecitabine 850 mg/m2 PO BID (XELOX)	S-l	High cholesterol	Angina-like chest pain + Sweating (x 2 episodes)	NSR, T wave inversion leads III and VI	CK300, MB 2.7, Troponin 0.25ng/ml	Moderate CAD with 40–50% disease of LCA, EF 55%, LVH	Toprol XL 25mg once daily Atorvastatin lomg once daily ASA 81 mg once daily	No

NA: Not Available; LCA: Left Coronary Artery; LVA: Left Ventricular Hypertrophy; HTN: Hypertension; CK: Creatine Kinase; LAD: Left Anterior Descending Artery; NSR: Normal Sinus Rhythm; LVEF: Left Ventricular Ejection Fraction; RCA: Right Common Aartery

## References

[1] Saif MW, Garcon MC, Rodriguez G, Rodriguez T. Bolus 5-fluorouracil as an alternative in patients with cardiotoxicity associated with infusion 5-fluorouracil and capecitabine: a case series. In Vivo. 2013 Jul 1;27(4):531–4. 23812226

[2] Vodenkova S, Buchler T, Cervena K, Veskrnova V, Vodicka P, Vymetalkova V. 5-fluorouracil and other fluoropyrimidines in colorectal cancer: Past, present and future. Pharmacology & Therapeutics. 2020 Feb 1;206:107447. 31756363 10.1016/j.pharmthera.2019.107447

[3] Thomas SA, Grami Z, Mehta S, Patel K. Adverse effects of 5-fluorouracil: focus on rare side effects. Cancer Cell & Microenvironment. 2016;3.

[4] Sara JD, Kaur J, Khodadadi R, Rehman M, Lobo R, Chakrabarti S, 5-fluorouracil and cardiotoxicity: a review. Therapeutic Advances in Medical Oncology. 2018 Jun 16;10:1758835918780140. 10.1177/1758835918780140 PMC6024329 29977352

[5] Saif W, Banchs J, Kohne C. Fluoropyrimidine-associated cardiotoxicity: incidence, clinical manifestations, mechanisms, and management. UpToDate. Waltham, MA: Wolters Kluwer Health, https://www.uptodate.com/login (2001, accessed 18 February 2018).

[6] Jensen SA, Sørensen JB. Risk factors and prevention of cardiotoxicity induced by 5-fluorouracil or capecitabine. Cancer Chemotherapy and Pharmacology. 2006 Oct 1;58(4):487–93. 16418875 10.1007/s00280-005-0178-1

[7] Papanastasopoulos P, Stebbing J. Molecular basis of 5-fluorouracil-related toxicity: lessons from clinical practice. Anticancer Research. 2014 Apr 1;34(4):1531–5. 24692679

[8] Saif MW, Smith M, Maloney A. The first case of severe Takotsubo cardiomyopathy associated with 5-fluorouracil in a patient with abnormalities of both dihydropyrimidine dehydrogenase (DPYD) and thymidylate synthase (TYMS) genes. Cureus. 2016 Sep;8(9). 10.7759/cureus.783 PMC5065345 27752409

[9] Deboever G, Hiltrop N, Cool M, Lambrecht G. Alternative Treatment Options in Colorectal Cancer Patients With 5-Fluorouracil-or Capecitabine-Induced Cardiotoxicity. Clinical Colorectal Cancer. 2013 Mar 1;12(1):8–14. 23102544 10.1016/j.clcc.2012.09.003

[10] Cunningham D, Pyrhönen S, James RD, Punt CJ, Hickish TF, Heikkila R, Randomised trial of irinotecan plus supportive care versus supportive care alone after fluorouracil failure for patients with metastatic colorectal cancer. The Lancet. 1998 Oct 31;352(9138):1413–8. 10.1016/S0140-6736(98)02309-5 9807987

[11] Sobrero AF, Maurel J, Fehrenbacher L, Scheithauer W, Abubakr YA, Lutz MP, EPIC: phase III trial of cetuximab plus irinotecan after fluoropyrimidine and oxaliplatin failure in patients with metastatic colorectal cancer. Journal of Clinical Oncology. 2008 May 10;26(14):2311–9. 18390971 10.1200/JCO.2007.13.1193

[12] Jonker DJ, O’Callaghan CJ, Karapetis CS, Zalcberg JR, Tu D, Au HJ, Cetuximab for the treatment of colorectal cancer. New England Journal of Medicine. 2007 Nov 15;357(20):2040–8. 18003960 10.1056/NEJMoa071834

[13] Van Cutsem E, Peeters M, Siena S, Humblet Y, Hendlisz A, Neyns B, Open-label phase III trial of panitumumab plus best supportive care compared with best supportive care alone in patients with chemotherapy-refractory metastatic colorectal cancer. Journal of Clinical Oncology. 2007 May 1;25(13):1658–64. 17470858 10.1200/JCO.2006.08.1620

[14] Grothey A, Van Cutsem E, Sobrero A, Siena S, Falcone A, Ychou M, Regorafenib monotherapy for previously treated metastatic colorectal cancer (CORRECT): an international, multicentre, randomised, placebo-controlled, phase 3 trial. The Lancet. 2013 Jan 26;381(9863):303–12. 10.1016/S0140-6736(12)61900-X 23177514

[15] Le DT, Uram JN, Wang H, Bartlett B, Kemberling H, Eyring A, Programmed death-1 blockade in mismatch repair deficient colorectal cancer.

[16] Chen J, Du F, Hu B, Chi C, Chu H, Jiang L, Severe cardiotoxicity in a patient with colorectal cancer treated with bevacizumab. Anticancer Research. 2017 Aug 1;37(8):4557–61. 28739752 10.21873/anticanres.11853

[17] Van Cutsem E, Cunningham D, Maroun J, Cervantes A, Glimelius B. Raltitrexed: current clinical status and future directions. Annals of Oncology. 2002 Apr 1;13(4):513–22. 12056700 10.1093/annonc/mdf054

[18] Ransom D, Wilson K, Fournier M, Simes RJ, Gebski V, Yip D, Final results of Australasian Gastrointestinal Trials Group ARCTIC study: an audit of raltitrexed for patients with cardiac toxicity induced by fluoropyrimidines. Annals of oncology. 2014 Jan 1;25(1):117–21. 24299960 10.1093/annonc/mdt479

[19] Iyer VN, Szybalski W. A molecular mechanism of mitomycin action: linking of complementary DNA strands. Proceedings of the National Academy of Sciences of the United States of America. 1963 Aug;50(2):355. 14060656 10.1073/pnas.50.2.355 PMC221180

[20] Saif MW, Kaley K, Brennan M, Garcon MC, Rodriguez G. Mitomycin-C and capecitabine (MIXE) as salvage treatment in patients with refractory metastatic colorectal cancer: a retrospective study. Anticancer Research. 2013 Jun 1;33(6):2743–6. 23749935

[21] Ferrarotto R, Machado K, Mak MP, Shah N, Takahashi TK, Costa FP, A multicenter, multinational analysis of mitomycin C in refractory metastatic colorectal cancer. European Journal of Cancer. 2012 Apr 1;48(6):820–6. 22330318 10.1016/j.ejca.2012.01.008

[22] Scheithauer W, Kornek GV, Brugger S, Ullrich-Pur H, Valencak J, Raderer M, Randomized phase II study of irinotecan plus mitomycin C vs. oxaliplatin plus mitomycin C in patients with advanced fluoropyrimidine/leucovorin-pretreated colorectal cancer. Cancer Investigation. 2002 Jan 1;20(1):60–8. 11853004 10.1081/cnv-120000367

[23] Chong G, Dickson JL, Cunningham D, Norman AR, Rao S, Hill ME, Capecitabine and mitomycin C as third-line therapy for patients with metastatic colorectal cancer resistant to fluorouracil and irinotecan. British Journal of Cancer. 2005 Sep;93(5):510–4. 16091760 10.1038/sj.bjc.6602733 PMC2361607

[24] Becouarn Y, Rougier P. Clinical efficacy of oxaliplatin monotherapy: phase II trials in advanced colorectal cancer. InSeminars in Oncology 1998 Apr (Vol. 25, No. 2 Suppl 5, pp. 23–31). 9609105

[25] Ezzeldin H, Okamoto Y, Johnson MR, Diasio RB. A high-throughput denaturing high-performance liquid chromatography method for the identification of variant alleles associated with dihydropyrimidine dehydrogenase deficiency. Analytical Biochemistry. 2002 Jul 1;306(1):63–73. 12069415 10.1006/abio.2002.5666

[26] Lopez CA, Azimi-Nekoo E, Chung SY, Newman J, Shen J, Saif MW. Meta-analysis and systematic review of the cardiotoxicity of TAS-102. Journal of Clinical Oncology. 2020;38:15_suppl, e16053–e16053.

[27] Mayer RJ, Van Cutsem E, Falcone A, Yoshino T, Garcia-Carbonero R, Mizunuma N, Randomized trial of TAS-102 for refractory metastatic colorectal cancer. New England Journal of Medicine. 2015 May 14;372(20):1909–19.. 25970050 10.1056/NEJMoa1414325

[28] Iyer VN, Szybalski W. Mitomycins and porfiromycin: chemical mechanism of activation and cross-linking of DNA. Science. 1964 Jul 3;145(3627):55–8. 14162693 10.1126/science.145.3627.55

[29] Cantrell JE Jr, Phillips TM, Schein PS. Carcinoma-associated hemolytic-uremic syndrome: a complication of mitomycin C chemotherapy. Journal of Clinical Oncology. 1985 May;3(5):723–34. 3923162 10.1200/JCO.1985.3.5.723

[30] Verweij J, Funke-Küpper AJ, Teule GJ, Pinedo HM. A prospective study on the dose dependency of cardiotoxicity induced by mitomycin C. Medical oncology and tumor pharmacotherapy. 1988 Sep 1;5(3):159–63. 3137399 10.1007/BF02986439

[31] Buzdar AU, Legha SS, Tashima CK, Hortobagyi GN, Yap HY, Krutchik AN, Adriamycin and mitomycin C: possible synergistic cardiotoxicity. Cancer Treatment Reports. 1978 Jul;62(7):1005–8. 688243

[32] Saif MW, Rosen LS, Saito K, Zergebel C, Ravage-Mass L, Mendelson DS. A phase I study evaluating the effect of CDHP as a component of S-1 on the pharmacokinetics of 5-fluorouracil. Anticancer Research. 2011 Feb 1;31(2):625–32. 21378348

[33] Franck C, Malfertheiner P, Venerito M. Safe administration of S-1 after 5-fluorouracil-induced cardiotoxicity in a patient with colorectal cancer. Case Reports. 2017 May 19;2017:bcr-2016. 10.1136/bcr-2016-219162 PMC5753671 28536214

[34] Kikuchi K, Majima S, Murakami M. Clinical survey on cardiotoxicity of tegafur (FT-207)--compilation of a nationwide survey. Gan to kagaku ryoho. Cancer & Chemotherapy. 1982 Aug;9(8):1482. 6820919

[35] Sorrentino MF, Kim J, Foderaro AE, Truesdell AG. 5-fluorouracil induced cardiotoxicity: review of the literature. Cardiology Journal. 2012;19(5):453–7. 23042307 10.5603/cj.2012.0084

[36] Saif MW, Shah MM, Shah AR. Fluoropyrimidine-associated cardiotoxicity: revisited. Expert Opinion on Drug Safety. 2009 Mar 1;8(2):191–202. 19309247 10.1517/14740330902733961

[37] Saif MW, Syrigos K, Mehra R, Mattison LK, Diasio RB. Dihydropyrimidine dehydrogenase deficiency (DPD) in GI malignancies: experience of 4-years. Pakistan Journal of Medical Sciences Quarterly. 2007;23(6):832. PMC2563423 18846242

[38] Saif MW, Diasio R. Is capecitabine safe in patients with gastrointestinal cancer and dihydropyrimidine dehydrogenase deficiency?. Clinical Colorectal Cancer. 2006 Jan 1;5(5):359–62. 16512996 10.3816/CCC.2006.n.007

[39] European Society of Medical Oncology. (2019, March 21). EMA Provides New Testing and Treatment Recommendations for Fluorouracil Capecitabine and Tegafur. Retrieved May 4, 2020, from https://www.esmo.org/oncology-news/ema-provides-new-testing-and-treatment-recommendations-for-fluorouracil-capecitabine-and-tegafur

